# Downregulation of miRNA-146a-5p promotes malignant transformation of mesenchymal stromal/stem cells by glioma stem-like cells

**DOI:** 10.18632/aging.103185

**Published:** 2020-05-25

**Authors:** Xingliang Dai, Yunfei Wang, Xuchen Dong, Minfeng Sheng, Haiyang Wang, Jia Shi, Yujing Sheng, Liang Liu, Qianqian Jiang, Yanming Chen, Bingshan Wu, Xuejun Yang, Hongwei Cheng, Chunsheng Kang, Jun Dong

**Affiliations:** 1 Brain Tumor Lab, Department of Neurosurgery, The Second Affiliated Hospital of Soochow University, Suzhou 215004, China; 2 Department of Neurosurgery, The First Affiliated Hospital of Anhui Medical University, Hefei 230022, China; 3 Department of Neurosurgery, Tianjin Medical University General Hospital, Tianjin 300052, China

**Keywords:** mesenchymal stromal/stem cell, glioma stem-like cell, miR-146a-5p, HNRNPD, dual-color fluorescence tracing

## Abstract

Mesenchymal stromal/stem cells (MSCs) are promising carriers in cell-based therapies against central nervous system diseases, and have been evaluated in various clinical trials in recent years. However, bone marrow-derived MSCs (BMSCs) are reportedly involved in tumorigenesis initiated by glioma stem-like cells (GSCs). We therefore established three different orthotopic models of GSC-MSC interactions *in vivo* using dual-color fluorescence tracing. Cell sorting and micropipetting techniques were used to obtain highly proliferative MSC monoclones from each model, and these cells were identified as transformed MSC lines 1, 2 and 3. Nineteen miRNAs were upregulated and 24 miRNAs were downregulated in all three transformed MSC lines compared to normal BMSCs. Reduced miR-146a-5p expression in the transformed MSCs was associated with their proliferation, malignant transformation and overexpression of heterogeneous nuclear ribonucleoprotein D. These findings suggest that downregulation of miR-146a-5p leads to overexpression of its target gene, heterogeneous nuclear ribonucleoprotein D, thereby promoting malignant transformation of MSCs during interactions with GSCs. Given the risk that MSCs will undergo malignant transformation in the glioma microenvironment, targeted glioma therapies employing MSCs as therapeutic carriers should be considered cautiously.

## INTRODUCTION

In the glioma microenvironment, there are not only glioma stem-like cell (GSC)-derived glioma cells, but also various kinds of tumor stromal cells, including peritumor astrocytes, microglia, fibroblasts, macrophages, dendritic cells, neutrophils, lymphocytes and endothelial cells [1]. Mesenchymal stromal/stem cells (MSCs) can migrate directly to the glioma microenvironment, and thus have been applied as antitumor delivery vectors for glioma treatment [1]. However, some studies have demonstrated that tumor stromal cells such as MSCs in the glioma microenvironment can not only promote tumor progression, but also enhance tumor resistance to chemotherapeutics [2]. Furthermore, several kinds of tumor stromal cells (e.g., fibroblasts, macrophages and oligodendrocytes) can undergo malignant transformation after interacting with cancer cells, especially cancer stem cells; indeed, our previous report indicated that the overexpression of telomerase reverse transcriptase was associated with GSC-induced MSC transformation [3]. However, the overexpression of telomerase reverse transcriptase in normal MSCs was only mildly tumorigenic *in vivo*, suggesting that other mechanisms contribute to MSC transformation during GSC-initiated tissue remodeling.

Certain pro-tumor genes can be horizontally transferred from tumor cells to stromal cells, thus malignantly transforming the stromal cells [4–6]; however, the chromosomes may be lost after fusion [8]. Glioma cells were reported to induce the transformation of MSCs *in vitro* without directly contacting them [7, 8], and the interleukin-6/signal transducer and activator of transcription 3 pathway was found to be involved in this process [9]. Granulocyte-macrophage colony-stimulating factor/interleukin-4 and soluble interleukin receptor/glycoprotein 130 may also contribute to MSC transformation *in vitro* [10, 11]. Simple long-term culture may induce the spontaneous malignant transformation of MSCs [12], but this finding has not been widely accepted as fact [13]. Bone marrow stromal cells in the rat brain were found to undergo malignant transformation *in vivo* in a tumor microenvironment containing tumor stem cell niches formed by orthotopically transplanted C6 glioma cells [14]; however, it is unclear where and how bone marrow stromal cells are transformed. In summary, the mechanisms responsible for the malignant transformation of MSCs in the glioma microenvironment have not been fully elucidated.

The aberrant expression of microRNAs (miRNAs), especially oncogenic or tumor suppressor miRNAs, promotes carcinogenesis, tumor progression, malignant transformation, tumor metastasis and anticancer treatment resistance [15–17]. High-throughput miRNA profiling techniques such as RNA sequencing and miRNA microarray analysis have greatly clarified the involvement of miRNAs in malignancies [18, 19]. Dysregulated miRNAs contribute to oncogenic transformation processes such as inflammation and metabolic reprogramming, thus creating a tumorigenic microenvironment that promotes the initiation and progression of neoplasms [20]. Altered miRNA expression profiles have been used to diagnose and stage various human tumors, and to predict their progression, prognosis and treatment response [21, 22]. However, further work is needed to determine the contributions of dysregulated miRNAs to the malignant transformation of MSCs, and to characterize the miRNA profiles of transformed MSCs in the glioma microenvironment.

In the current study, we established three different *in vivo* GSC-MSC interaction models so that we could observe the morphological and functional changes of MSCs that had interacted with GSCs. We then used RNA sequencing to analyze the miRNA profiles of the transformed MSCs, and examined the involvement of miR-146a-5p in MSC transformation both *in vitro* and *in vivo*. This study has provided novel evidence on the molecular mechanisms underlying the malignant transformation of bone marrow-derived MSCs (BMSCs), illustrating the potential risks of MSC-based clinical trials for glioma.

## RESULTS

### Biomarkers of MSCs were associated with the survival of glioma patients

**Figure 1:**
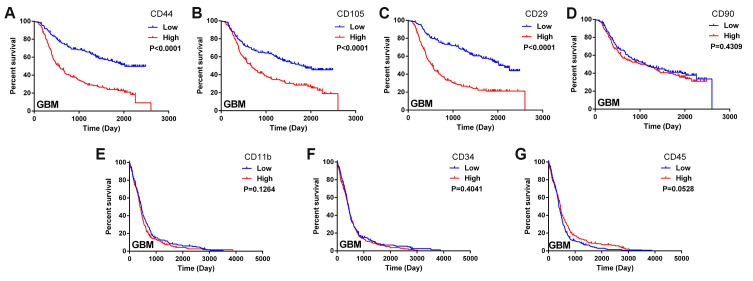
**Biomarkers of MSCs in the glioma microenvironment.** (**A**–**D**) Kaplan-Meier survival curves based on the expression of the MSC biomarkers CD44, CD105, CD29 and CD90 in glioblastoma tissues from the CGGA dataset; (**E**–**G**) Kaplan-Meier survival curves based on the expression of the MSC biomarkers CD11b, CD34 and CD45 in glioblastoma tissues from TCGA.

In this study, we obtained data on glioma patients from the databases of the Chinese Glioma Genome Atlas (CGGA) and The Cancer Genome Atlas (TCGA). We constructed Kaplan-Meier survival curves and performed a log-rank comparison to analyze the correlations between MSC surface marker levels in glioma tissues and overall glioma patient survival. The expression of the positive MSC markers CD44, CD105 and CD29 exhibited a significant inverse correlation with the prognosis of glioma patients (Figure 1A–1C). Another positive marker, CD90, was not significantly associated with overall survival (Figure 1D). Additionally, the expression of the negative MSC markers CD11b, CD34 and CD45 did not correlate significantly with the prognosis of glioma patients (Figure 1E–1G). These results suggested that higher MSC marker expression was associated with a shorter survival period and a poorer prognosis in glioma patients.

### Preparation of the dual-color fluorescence-tracing intracranial GSC-MSC interaction model

**Figure 2:**
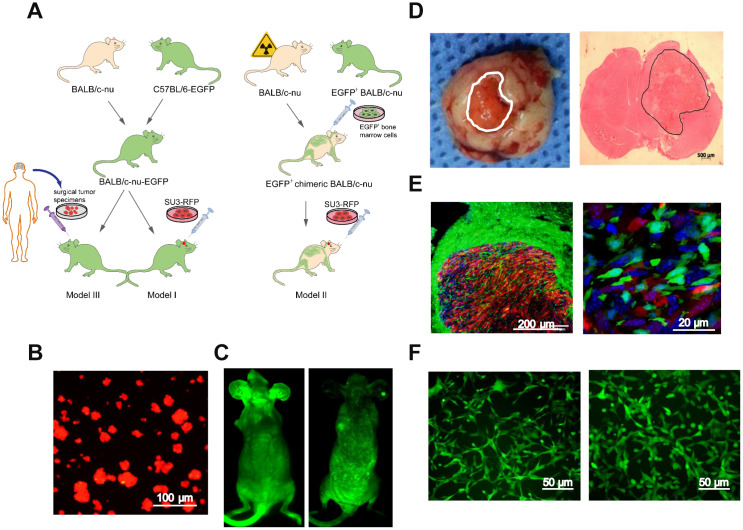
**Intracerebral xenografting of GSCs.** (**A**) Schematic overview of the establishment of the dual-color fluorescence-tracing intracranial GSC-MSC interaction model; (**B**) SU3-RFP cells; (**C**) Transgenic EGFP^+^ nude mouse, and chimeric nude mice with autologous bone marrow deprivation and exogenous EGFP^+^ bone marrow cell transplantation; (**D**) Transplanted tumor observed under natural light, and H&E staining of a transplanted tumor section; (**E**) Transplanted tumor from an EGFP^+^ nude mouse under a fluorescence microscope; (**F**) Monocloned EGFP^+^ cells with high proliferative capacities, TMEC1, TMEC2. Scale bars: (**A**) 100 μm; (**C**) 500 μm; (**D**) 200 μm; (**F**) 50 μm.

Next, we generated GSC-MSC interaction models, as shown in the schematic diagrams in Figure 2A. Human GSCs (SU3 cells) were stably transfected with the red fluorescent protein (*RFP*) gene, and the SU3-RFP cells displayed 100% RFP expression (Figure 2B). We also generated transgenic enhanced green fluorescent protein (EGFP)^+^ BALB/c athymic nude mice, which expressed EGFP ubiquitously, except in enucleated cells (Figure 2C, left). To generate chimeric BALB/c nude mice, we used X-ray irradiation to autologously deplete the bone marrow function of normal BALB/c nude mice, and reconstructed their bone marrow by injecting their tail veins with exogenous EGFP^+^ bone marrow cells harvested from the EGFP^+^ BALB/c athymic nude mice; thus, in the chimeric mice, only bone marrow-derived cells expressed EGFP (Figure 2C, right).

An SU3-RFP cell suspension was injected into the cerebral caudate of the EGFP^+^ nude mice (Model I) and the chimeric mice (Model II) for approximately 35 days. Then, tumor-bearing mice from both models were sacrificed, and the intracranial tumors were examined under natural light and after hematoxylin and eosin (H&E) staining (Figure 2D). SU3-derived RFP^+^ tumor cells and EGFP^+^ host cells in the tumor sections could be distinguished under fluorescence microscopy (Figure 2E). Under confocal microscopy, it was evident that GSCs and MSCs directly contacted each other within the transplanted tumors (white arrow, Supplementary Figure 1). Flow cytometry (MoFlo XDP; Beckman Coulter, USA) was used to sort cells expressing EGFP, which were detected at percentages of 5.46% (Model I) and 8.64% (Model II) (Supplementary Figure 2A, 2B). A micropipetting technique was used to obtain monoclones of EGFP-expressing cells with high proliferative capacities from Model I (tumor microenvironment cells 1, TMEC1) and Model II (TMEC2) (Figure 2F).

**Figure 3:**
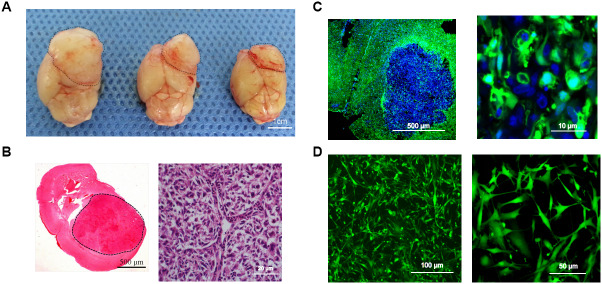
**Intracerebral-xenograft-derived glioblastoma specimens.** (**A**) Whole brain of a tumor-bearing mouse; (**B**) H&E staining; (**C**) Transplanted tumor viewed under a fluorescence microscope; (**D**) Monocloned EGFP^+^ TMEC3 cells. Scale bars: (**B**) 500 μm (left), 20 μm (right); (**C**) 10 μm; (**D**) 100 μm (left), 50 μm (right).

Surgical tumor specimens from a 34-year-old male patient diagnosed with glioblastoma were transplanted into the caudate of EGFP^+^ nude mice via a stereotactic trocar system (1-mm^3^ specimens, Supplementary Figure 2C). After the mice were sacrificed, their tumors were observed under natural light (Figure 3A). H&E staining of xenograft sections revealed densely arranged tumor cells (Figure 3B), and fluorescence microscopy revealed an irregular round tumor border enclosing some EGFP^+^ cells (Figure 3C). After primary culture of the transplanted tumors, cells expressing EGFP were sorted, and were found to account for 29.25% of the total cells (Supplementary Figure 2D). From the tumor model based on the transplantation of surgical glioblastoma specimens (Model III), one EGFP^+^ cell line (TMEC3) was cloned (Figure 3D).

As a control experiment, we performed direct co-culture *in vitro* to assess whether GSCs directly interacted with MSCs. Using time-lapse photography of a living cell workstation, we did indeed observe interactions, including direct contact, between GSCs and BMSCs. We even detected the exchange of cytoplasmic substances between the cells, both through direct contact points (black arrow, Supplementary Figure 3) and through slender tubular structures (black arrow, Supplementary Figure 4) that turned yellow after the intercellular cytoplasm exchange (white arrow, Supplementary Figure 4). However, when GSCs and MSCs were indirectly co-cultured in a Transwell system *in vitro*, no significant changes were observed in the growth characteristics of the MSCs (Supplementary Figure 5).

### Malignant transformation of BMSCs

**Figure 4:**
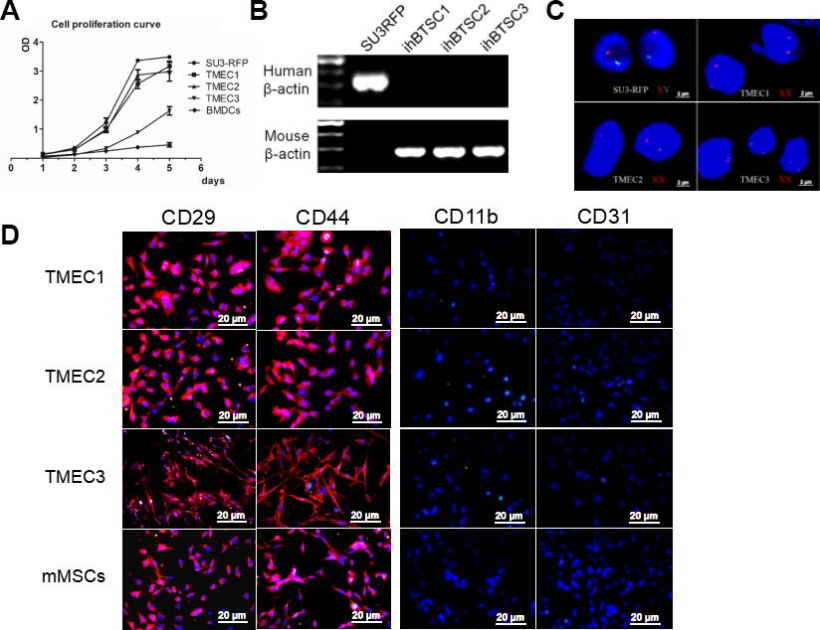
**Transformation of host stromal cells.** (**A**) Cell proliferation curve; (**B**) RT-PCR analysis of human and mouse *β-actin* expression in SU3 cells and three TMEC lines; (**C**) FISH assay of chromosomes in SU3 cells and transformed cells; (**D**) Immunofluorescence of the three tMSC lines. Scale bars: (**C**) 2 μm; (**D**) 20 μm.

All three cloned EGFP^+^ cell lines (TMEC1, TMEC2 and TMEC3) exhibited strong proliferation abilities *in vitro*, and their cell expansion activities were much higher than those of normal bone marrow cells, but lower than those of SU3 cells (Figure 4A). TMEC1 and TMEC2 cells exhibited high tumorigenicity 25 days after being subcutaneously transplanted (1×10^6^ cells) into BALB/c nude mice; both cell lines had a tumor formation rate of 100% (5/5). However, subcutaneously transplanted TMEC3 cells did not form tumors in any of the five mice (Supplementary Figure 2E).

The three transformed cell lines expressed mouse *β-actin,* but not human *β-actin* (Figure 4B). A fluorescence in situ hybridization (FISH) assay of the sex chromosomes revealed that the karyotype of the SU3 cells was XY (X, red fluorescent probe; Y, green fluorescent probe) (Figure 4C), in accordance with clinical data showing that SU3 cells were derived from a male patient [23, 24]. The karyotypes of all three transformed cell lines were XX, consistent with the karyotypes of the female host mice (Figure 4C). Immunofluorescence assays demonstrated that TMEC1, TMEC2 and TMEC3 cells expressed biomarkers of MSCs, as the cells were positive for CD29, CD44, CD105 and CD90 but negative for CD11b, CD31, CD34 and CD45. Normal MSCs were used as the positive control (Figure 4D and Supplementary Figure 2F).

**Figure 5:**
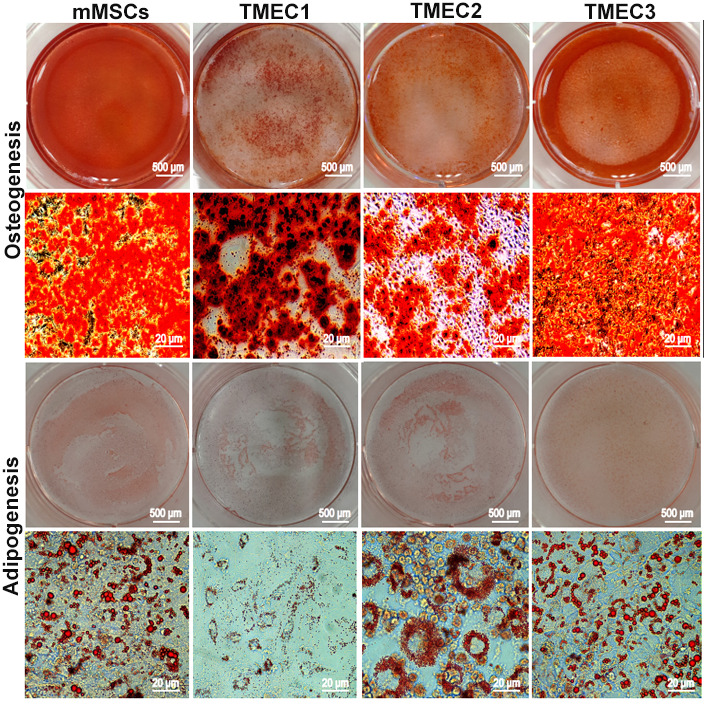
**Osteogenic and adipogenic differentiation assays in tMSCs.** Scale bars: 20 μm.

We then performed an osteogenic differentiation assay, and found that mineralized nodules emerged from all three cell lines after seven days of differentiation induction. When we conducted an adipogenic differentiation assay, small fat droplets gradually coalesced into large fat droplets after seven days of differentiation induction, and brown adipose tissue deposition occurred after 14 days of *in vitro* induction. These multidirectional differentiation assays confirmed that TMEC1, TMEC2 and TMEC3 cells were transformed BMSCs and still had differentiation potential. Specifically, TMEC1 cells exhibited obvious osteogenic and slight adipogenic differentiation potential, TMEC2 cells exhibited notable adipogenic differentiation potential, and TMEC3 cells exhibited strong osteogenic and adipogenic differentiation potential, similar to those of normal MSCs (Figure 5). These data suggested that the three transformed EGFP-expressing TMEC lines from the different models were BMSCs; thus, these cells were named transformed MSCs (tMSC1, tMSC2 and tMSC3 for Models I, II and III, respectively).

### Differentially expressed miRNAs in tMSCs

**Figure 6:**
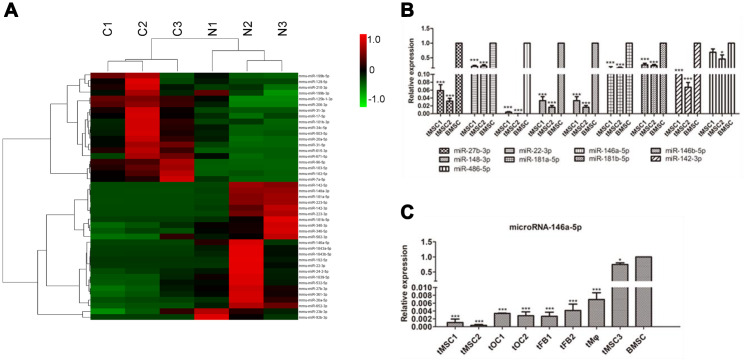
**MiRNA profiles of tMSCs.** (**A**) Differentially expressed miRNAs; (**B**) Verification of miRNAs identified in sequencing experiments; (**C**) Expression of miRNA-146a-5p in tMSCs compared with normal BMSCs. * P<0.05, *** P<0.001.

We then performed small RNA sequencing on the tMSCs and normal BMSCs. We found that 19 miRNAs were significantly upregulated and 24 miRNAs were significantly downregulated in tMSCs (n=3) compared with normal BMSCs (n=3) (Figure 6A). Quantitative real-time PCR (qRT-PCR) was used to further assess nine of these 43 miRNAs (Figure 6B, 6C). Because the tMSC1 and tMSC2 cells were highly proliferative and exhibited strong tumorigenicity, we used these cells to examine the correlations between miRNA levels and biological functions contributing to malignant transformation. The qRT-PCR analysis results for the nine selected miRNAs were consistent with the RNA sequencing results.

We found that miR-146a-5p expression was reduced in all three tMSC lines compared with normal MSCs (927.99-fold in tMSC1, 2849.74-fold in tMSC2 and 1.25-fold in tMSC3). Interestingly, miR-146a-5p was significantly downregulated in the tMSCs with higher tumorigenic abilities (tMSC1 and tMSC2), but only slightly downregulated in those with a lower tumorigenic ability (tMSC3 cells) (Figure 6C). These data suggested that lower miR-146a-5p expression may be associated with MSC transformation and tumorigenicity.

### Overexpression of miR-146a-5p partially reversed the malignant phenotype of tMSCs

**Figure 7:**
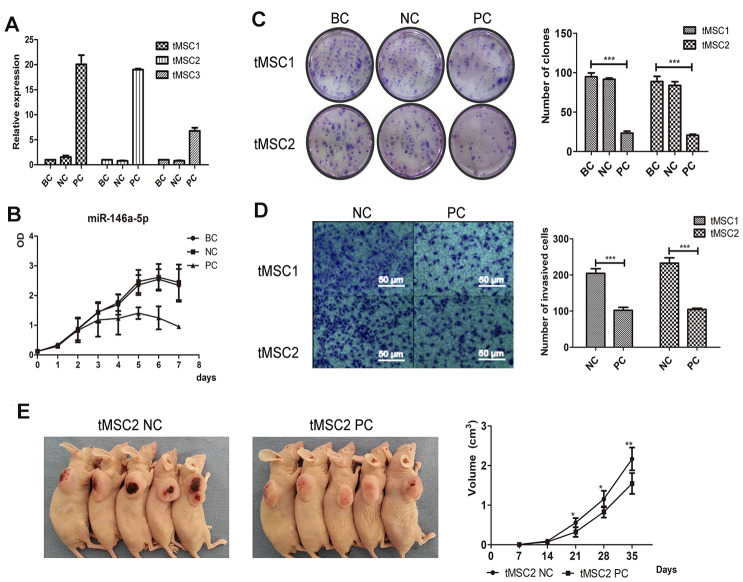
**Overexpression of miR-146a-5p partially reversed the malignant phenotype of tMSCs.** (**A**) Relative expression of miR-146a-5p after lentiviral vector transfection; (**B**) Cell proliferation activity of miR-146a-5p-overexpressing, negative control (NC) and blank control (BC) tMSC1 cells; (**C**) Colony formation assays of BC, NC and miR-146a-5p-overexpressing tMSC1 and tMSC2 cells; (**D**) Invasion assays of NC and miR-146a-5p-overexpressing tMSC1 and tMSC2 cells; (**E**) Tumorigenicity of NC and miR-146a-5p-overexpressing tMSC1 cells. * P<0.05, ** P<0.01, *** P<0.001.

Next, we used lentiviral vectors to overexpress miR-146a-5p in the three tMSC lines. The transfection induced miR-146a-5p expression 20.06-fold, 19.02-fold and 6.79-fold in tMSC1, tMSC2 and tMSC3 cells, respectively (Figure 7A). Cell proliferation was significantly lower in miR-146a-5p-overexpressing tMSC1 (PC) cells than in the blank control (BC) and negative control (NC) cells (Figure 7B). Overexpression of miR-146a-5p in tMSC1 and tMSC2 cells reduced their colony formation abilities in a colony formation assay (P<0.001); the colony numbers of blank control, negative control and miR-146a-5p-overexpressing tMSC1 cells were 94.75±4.32, 91.50±1.50 and 23.25±2.17, while those of blank control, negative control and miR-146a-5p-overexpressing tMSC2 cells were 88.75±5.63, 83.75±4.15 and 20.50±1.12, respectively (Figure 7C). The invasion abilities of tMSC1 and tMSC2 cells also decreased significantly after miR-146a-5p overexpression (P<0.001) (Figure 7D). The tumors generated from miR-146a-5p-overexpressing tMSC1 cells were appreciably smaller in volume than the tumors generated from control cells 28 days after their subcutaneous transplantation into BALB/c nude mice (Figure 7E).

### HNRNPD is a direct target gene of miR-146a-5p

**Figure 8:**
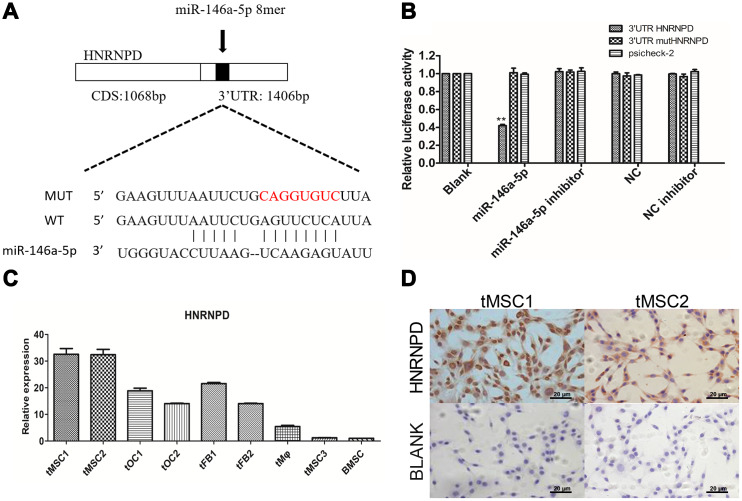
**Expression of the target gene *HNRNPD* in tMSCs.** (**A**) Putative miR-146a-5p target sites in the *HNRNPD* 3'UTR; (**B**) Dual-luciferase assay; (**C**) qRT-PCR; (**D**) Immunocytochemistry of tMSC1 and tMSC2 cells. ** P<0.01.

We then performed target gene prediction on the nine differentially expressed miRNAs validated in this study, and conducted Gene Ontology (GO) analyses (top 30 shown in Supplementary Figure 8A) and Kyoto Encyclopedia of Genes and Genomes (KEGG) pathway analyses (top 20 shown in Supplementary Figure 8B). The TargetScan prediction database identified heterogeneous nuclear ribonucleoprotein D (*HNRNPD*) as a target gene of miR-146a-5p (Figure 8A). In a luciferase reporter gene assay, miR-146a-5p significantly repressed the expression of *HNRNPD* by binding to its 3' untranslated region (UTR) (P<0.01). Luciferase activity was not detected in the negative control group (containing a mutated 3'UTR of *HNRNPD*) or the blank group, suggesting that miR-146a-5p specifically binds to the 3'UTR of *HNRNPD* (Figure 8B). *HNRNPD* mRNA was significantly upregulated in tMSC1 and tMSC2 cells compared with BMSCs (Figure 8C), but was not highly expressed in tMSC3 cells. Immunocytochemical staining of tMSC1 and tMSC2 cells further verified the high expression of HNRNPD at the protein level, consistent with the transcriptional results (Figure 8D).

### HNRNPD contributed to the malignant transformation of MSCs

**Figure 9:**
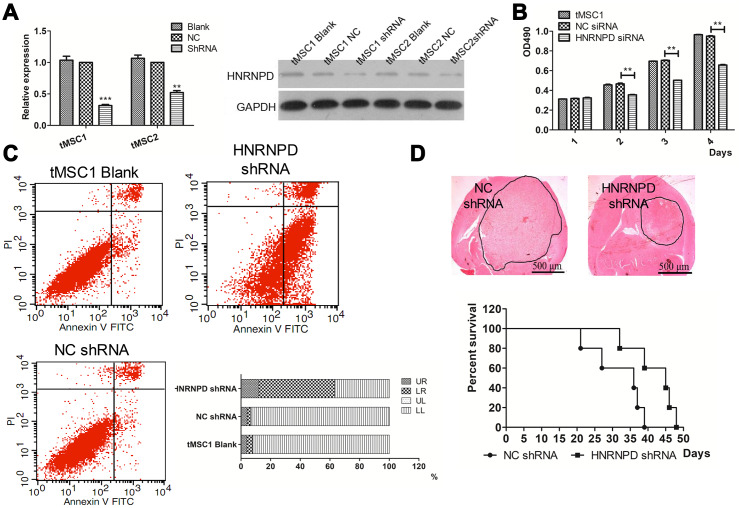
**Correlation of HNRNPD expression with MSC transformation.** (**A**) Protein expression after siRNA interference; (**B**) Cell proliferation; (**C**) Apoptosis; (**D**) Intracranial transplantation and survival time. ** P<0.01.

To further study the involvement of HNRNPD in the transformation of MSCs, we used small interfering RNA (siRNA) to downregulate *HNRNPD* in tMSC1 cells. Western blotting confirmed that HNRNPD protein expression was downregulated in the siRNA-treated cells (Figure 9A). The siRNA also significantly inhibited cell proliferation, as shown in an MTS assay (Figure 9B).

Flow cytometry was then used to assess changes in apoptosis and the cell cycle. The average total percentage of apoptotic cells was 63.06±3.48% in the siRNA-treated group, which was significantly higher than that in the control (6.73±0.52%) and blank control (7.76±0.99%) groups (P<0.01, Figure 9C). In a colony formation assay, the colony formation rate of siRNA-treated tMSC1 clones was 51±7.0%, which was significantly lower than the rates of the control (94.33±1.89%) and blank control (95.33±2.05%) groups (P<0.01, Supplementary Figure 6A). Significant cell cycle arrest was observed in G1 phase, and occurred in a higher proportion of siRNA-treated cells (61.01±1.22%) than control (54.58±0.97%) or blank control (53.88±1.04%) cells (P<0.01, Supplementary Figure 6B).

We also intracranially inoculated mice with tMSC1 cells that were stably transfected with *HNRNPD* siRNA. The tumor volume was significantly smaller and the median survival time was nine days longer in the siRNA-treated group than in the control group (Figure 9D). Thus, the results of *HNRNPD* downregulation were similar to those of miR-146a-5p overexpression.

To assess whether the downregulation of miR-146-5p promoted the malignant transformation of tMSCs by inducing *HNRNPD* expression, we overexpressed miR-146a-5p, HNRNPD or both in tMSC1 cells. The proliferation curve and clone formation results demonstrated that tMSC1 cell proliferation was significantly inhibited in the miR-146a-5p overexpression group and significantly elevated in the HNRNPD overexpression group. In the group overexpressing both miR-146a-5p and HNRNPD, the proliferation of tMSC1 cells was higher than that of the control group, indicating that the ability of miR-146a-5p to inhibit tMSC proliferation depended on its ability to downregulate *HNRNPD* (Supplementary Figure 7).

In conclusion, deficiency of miR-146a-5p led to the overexpression of its target gene *HNRNPD*, thereby promoting the malignant transformation of MSCs.

## DISCUSSION

Our dual-color fluorescence-tracing GSC-MSC interaction models revealed the infiltration of certain host stromal cells into xenograft tumors (Figure 1E, 2C). In frozen tumor sections from Model II, many exogenous bone marrow-derived cells expressing EGFP were observed, suggesting that host BMSCs can migrate from the bone marrow to the tumor parenchyma and undergo malignant transformation in the glioma microenvironment. The malignant transformation of MSCs has been reported previously [25], but the malignant transformation of MSCs recruited from the bone marrow to the glioma microenvironment has never been reported. Our model was an effective visual platform for observing tumor-stromal interactions, and thus was an excellent tool for studying the interactions between GSCs and infiltrating MSCs.

MiRNA sequencing allowed us to identify miRNAs that were differentially expressed between normal MSCs and tMSCs. The abnormal expression of these miRNAs was further verified in a qRT-PCR assay, and miR-146a-5p was found to be significantly downregulated in several kinds of transformed TMECs. However, miR-146a-5p has divergent functions in different types of tumors. For instance, miR-146a-5p functions as an oncogene when it is overexpressed in oral tumors, cervical cancer cells and papillary thyroid carcinomas, but serves as a tumor suppressor in triple-negative breast cancer, colon carcinoma, non-small cell lung cancer cells and prostate cancer [26]. Zhao et al. [27] found that knocking out miR-146a in C57BL/6 mice led to histologically and immunophenotypically defined myeloid sarcomas, and also resulted in some lymphomas with true malignancies. Contreras et al. [28] reported that miR-146a deficiency contributed to B-cell oncogenesis by inducing the *early growth response-1* gene. The functions of miR-146a in malignant hematopoietic stem cells have also been studied [29]. We observed that the malignant transformation of BMSCs was associated with the downregulation of miR-146a-5p due to the resulting overexpression of *HNRNPD*.

Changes in HNRNPD expression are involved in the development and progression of multiple types of solid tumors. Gouble et al. [30] examined transgenic mice that overexpressed p37^AUF1^ (HNRNPD), and found that they overexpressed the mRNAs for multiple tumor-associated genes, including *c-Myc*, *c-Jun*, *c-Fos*, *granulocyte macrophage colony-stimulating factor*, *tumor necrosis factor-α* and *cyclin D1*, resulting in the spontaneous formation of soft tissue sarcoma. In another study, *HNRNPD* mRNA levels in tumor tissues and corresponding paracancerous tissues were compared in 154 patients with 19 kinds of cancers. *HNRNPD* was significantly upregulated in 13% of the tumors, consistent with other reports of its upregulation in esophageal, breast, skin, thyroid and liver cancers [31, 32].

In this study, the binding between miRNA-146a-5p and *HNRNPD* was predicted in a bioinformatic assay and confirmed in a dual luciferase assay. Suppressing the expression of HNRNPD significantly inhibited cell cycle progression and proliferation *in vitro*, and also increased apoptosis, inhibited tumor cell proliferation and prolonged survival in tumor-bearing mice. Our results suggested that the downregulation of miRNA-146a-5p directly enhances the malignant characteristics of tMSCs by upregulating *HNRNPD*.

MSCs are capable of infiltrating the glioma microenvironment, and thus can be applied as carriers of anticancer molecules targeting glioma cells; however, the fate of MSCs after their homing to gliomas remains largely unknown. According to our analyses of the CGGA and TCGA databases, the levels of the MSC biomarkers CD29, CD44 and CD105 were associated with the prognosis of glioma patients. CD44 expression was previously reported to promote BMSC migration toward gliomas [33]. Other studies [34, 35] have noted that, compared with CD90^+^ MSCs, CD90^-^ MSCs promote tumor vascularization and immunosuppression, which may explain why there was no significant difference in glioma patient survival according to CD90 expression in our study. The *in vitro* and *in vivo* experiments in this paper confirmed that BMSCs migrated to the glioma microenvironment and were malignantly transformed after interacting with GSCs. The downregulation of miRNA-146a-5p promoted GSC-initiated MSC transformation by derepressing the *HNRNPD* gene.

In summary, BMSCs isolated from intracerebral xenograft GSC tumors were found to undergo malignant transformation, and the suppression of miR-146a-5p was found to promote this process by activating the oncogene *HNRNPD*. These results suggest that targeted glioma therapies using MSCs as cellular carriers should be used cautiously, due to the risk that MSCs will undergo malignant transformation in the glioma microenvironment.

## MATERIALS AND METHODS

### Correlation studies between MSC surface marker expression and glioma patients’ prognostic information

We analyzed the association of positive (CD29, CD44, CD105 and CD90) and negative (CD11b, CD31, CD34 and CD45) MSC marker expression with glioma prognosis in both the CGGA and TCGA databases. Kaplan-Meier survival analysis was used to estimate the survival distributions, and the log-rank test was used to assess the statistical significance of survival differences between groups stratified based on the median value of each marker. Pearson correlation was used to determine the significance of differences, and t-tests were used to determine the significance of two-group comparisons. Data are presented as the mean ± standard error. A two-sided P value < 0.05 was considered significant.

### Cell lines and animals

We established the RFP-expressing GSC line SU3-RFP by using a lentiviral vector to stably transfect SU3 cells with the *RFP* gene, as previously described [23, 24]. Cells were cultured in Dulbecco’s modified Eagle’s medium (DMEM)/F12 medium (Gibco, USA) containing 20 ng/mL basic fibroblast growth factor (Gibco) and 20 ng/mL epidermal growth factor (Gibco).

To establish ubiquitous EGFP expression in athymic BALB/c nude mice, we crossed BALB/c nude mice with C57BL/6-EGFP mice [36]. BALB/c nude mice expressing EGFP only in their bone marrow-derived cells (named chimeric BALB/c nude mice) were established through bone marrow damage/reconstruction technology. Briefly, X-ray radiation was used to destroy the bone marrow function of normal BALB/c nude mice, and the mice were then transplanted (via tail vein injection) with 1×10^7^ EGFP^+^ bone marrow-derived cells harvested from EGFP^+^ BALB/c nude mice. Female mice were selected for the experiment, and were raised in the specific-pathogen-free experimental animal center of Soochow University. The animal studies and clinical tumor specimen collection were conducted with the approval and permission of the Soochow University Ethics Committee. Informed consent was obtained prior to sample acquisition.

### Orthotopic transplantation

We used previously described techniques to perform intracranial orthotopic tumor transplantation [37]. Briefly, a Hamilton syringe was used with stereotactic technique to slowly inject a suspension of SU3-RFP cells (1×10^5^) in 15 μL of DMEM into the cerebral caudate of EGFP^+^ BALB/c nude mice (n=3, Model I) or chimeric BALB/c nude mice expressing EGFP only in their bone marrow-derived cells (n=3, Model II). For clinical tumor specimens, small fragments (approximately 1 mm^3^ each) of fresh surgical tumor specimens were transplanted into the cerebral caudate of EGFP^+^ BALB/c nude mice (n=3, Model III) via manual manipulation with a microinjection trocar system developed in-house (Chinese Patent, CN 101569564A). Tumor specimens were obtained with informed consent from a 34-year-old male patient diagnosed with glioblastoma (World Health Organization grade IV). The tumor exhibited O6-methylguanine-DNA methyltransferase promoter methylation, low O6-methylguanine-DNA methyltransferase expression and wild-type isocitrate dehydrogenase 1/2 expression.

### Time-lapse photography in vitro

GSCs and MSCs were co-cultured *in vitro* in a living cell workstation and were imaged via time-lapse photography. Cell suspensions of GSCs and MSCs were made, and the cells were directly co-cultured at a ratio of 1:8 (GSCs : MSCs). The cell culture parameters were adjusted at the living cell workstation, the appropriate observation field was selected, and pictures were taken every 10 minutes during 96 hours of continuous culture.

### Subculturing, sorting and cloning of tumor stromal cells

Tumor-bearing mice were sacrificed approximately 35 days after orthotopic xenotransplantation. After the intracranial xenograft tumors were harvested, pieces of the tumors were dissociated and subcultured *in vitro*, and other pieces were used for pathological examination. Frozen sections were stained with H&E, and 4',6-diamidino-2-phenylindole (KeyGen, Nanjing, China) was used for nuclear labeling. The sections were then observed under both a fluorescence microscope and a confocal laser scanning microscope (ZEISS, Germany).

For cell subculture, minced tumor tissue was washed with cold phosphate-buffered saline, digested with 0.02% trypsin and cultured in DMEM containing 10% fetal bovine serum (FBS, Gibco). After the cells had been serially subcultivated *in vitro* for approximately two weeks, fluorescence-activated cell sorting was used to sort suspensions of RFP^+^ tumor cells and EGFP^+^ stromal cells, and EGFP-expressing tumor stromal cells were collected. Then, the limiting dilution method and single-cell cloning techniques were used to re-purify EGFP-expressing cells with high proliferation abilities from the different animal models [23, 38]. The EGFP-expressing tumor stromal cells from Models I, II and III were named EGFP^+^ tumor microenvironment cells 1 (^EGFP+^TMEC1, ^EGFP+^TMEC2 and ^EGFP+^TMEC3, respectively).

### Characterization and analysis of highly proliferative EGFP^+^ stromal cells

The proliferation of the TMECs (TMEC1, TMEC2 and TMEC3) was analyzed with a Cell Counting Kit-8 (CCK-8; Dojindo, Kumamoto, Japan) according to the manufacturer’s instructions. Primers specific for human and mouse *β-actin* were used for reverse transcription polymerase chain reaction (RT-PCR) analyses. The primers are listed in Supplementary Table 1. Human and mouse sex-specific FISH assays were performed to analyze whether the EGFP^+^ TMECs were of mouse or human origin, according to a method described previously [10]. To evaluate the tumor formation ability of the EGFP^+^ TMEC monoclones, we subcutaneously inoculated the right forelimbs of BALB/c nude mice with the cells (1x10^6^ cells/mouse).

An immunofluorescence assay was used to identify cell surface markers. Antibodies against CD29, CD44, CD105, CD90, CD11b, CD31, CD34 and CD45 (Abcam, UK) were applied to detect the expression of the relevant surface markers, in accordance with the manufacturer’s instructions. Cy3-labeled goat anti-rabbit or anti-rat IgG (Beyotime, Shanghai, China) was applied as a secondary antibody. The expression of the cell surface markers was observed under a fluorescence microscope (Olympus IX51; Tokyo, Japan), and images were acquired and merged in the software in accordance with the manufacturer’s instructions.

Osteogenic and adipogenic differentiation assays were performed to evaluate the potential for pluripotent differentiation. Cells were plated in six-well plates (coated with 0.1% gelatin) at a density of 5×10^5^ cells/well in DMEM medium. After 24 h, the medium was replaced with a conditioned medium designed to induce osteogenic differentiation (Cyagen, Guangzhou, China). The medium was replaced every other day. Specific staining was used to assess differentiation (Alizarin red staining for osteogenic differentiation and Oil-Red-O staining for adipogenic differentiation) on days 7, 14 and 21. D1 ORL UVA mouse MSCs (American Type Culture Collection, P4) were used as the positive control.

### Small RNA sequencing and identification of differentially expressed miRNAs

RNA was extracted with a mirVana^TM^ miRNA Isolation Kit (Ambion, Austin, TX, USA) according to the manufacturer's instructions. The RNA quality was evaluated with an Agilent 2100 Bioanalyzer (Agilent Technologies, USA). Total RNA (10 μg) was used to construct a small RNA sequencing library according to the Illumina miRNA sample preparation protocol. As we have previously reported [39], polyacrylamide gel electrophoresis was used to purify and enrich RNA (16-30 nucleotides). Then, the 5' and 3' termini of the RNA were ligated to proprietary adaptors, and the RNA was transcribed to cDNA. Small RNA sequencing was performed with the high-throughput sequencing technology developed by Illumina.

The sequence annotation, data analysis and screening of differentially expressed miRNAs were performed in accordance with the manufacturer’s manual. Briefly, after the adaptors and low-quality sequences had been filtered out, BLAST (version 2.2.11) was used to map the reads against all the annotated mature miRNA sequences in mice (Sanger miRBase 17.0). The miRNA sequence reads were normalized as reads per million (RPM) values according to the following formula: RPM = (normalized reads/total miRNA matches) x 1,000,000. MiRNAs were deemed to be significantly differentially expressed if the RPM was >2 or <0.5.

### qRT-PCR analysis of miRNA and target genes

To verify the sequencing data, we selected eight differentially expressed miRNAs (miR-27b-3p, miR-22-3p, miR-146a-5p, miR-146b-5p, miR-148-3p, miR-181a-5p, miR-181b-5p and miR-142-3p) and one non-differentially expressed miRNA (miR-486-5p) for qRT-PCR validation. The PCR Universal Reagents and primers specific for the mature miRNAs and U6 snRNA were purchased from GenePharma (Suzhou, China). An ABI StepOne system (Applied Biosystems, Foster City, CA, USA) was used to perform the qRT-PCR.

Transcript-level target gene quantification was also performed via qRT-PCR. The primers (Supplementary Table 1) were designed and synthesized by Genewiz Biotech (Suzhou, China). Each sample was analyzed in triplicate, and the experiment was repeated three times. The miRNA and mRNA levels were normalized to those of snRNA U6 and *β-actin*, respectively, and the 2^-ΔΔCt^ method was used for quantification.

### MiRNA-146a-5p overexpression

To establish an EGFP^+^ TMEC line overexpressing mature miR-146a-5p miRNA, we used pGLV10/U6/RFP/Puro lentiviral vectors containing miR-146a-5p sequences (generated by GenePharma, Suzhou, China). The empty pGLV10/U6/RFP/Puro vector (G05AZ, GenePharma) was used as the control. An infection assay was performed with biohazard safety equipment, in accordance with the manufacturer’s instructions. Stable cell lines were selected with 10 μg/mL puromycin.

### Invasion and colony formation assays in vitro

A cell invasion assay was performed with Transwell chambers (Corning, NY, USA), as described previously [38]. Briefly, the upper surface of the chamber was coated with a thin layer of Matrigel matrix (BD Bioscience). Cells (3×10^4^) in 120 μL of DMEM without FBS were seeded into the upper chambers, and the lower chambers were filled with DMEM containing 20% FBS. After incubation at 37.0 °C for 36 hours, the upper chambers were removed and fixed with 4% paraformaldehyde. The cells that had migrated to the lower surface were stained with 0.1% crystal violet and counted under a microscope (Olympus IX51, Japan).

For the colony formation assay, cells were diluted to 200 cells/mL in DMEM containing 10% FBS, and were added to each well of a six-well plate. After two weeks of culture, the cell colonies were fixed with 4% paraformaldehyde and stained with Giemsa (Solarbio, Shanghai, China).

### Tumorigenicity assay

To compare the *in vivo* tumor formation capabilities of transformed cells with or without miR-146a-5p overexpression, we subcutaneously injected 1x10^6^ TMEC2 cells, TMEC2 cells expressing the negative control vector or TMEC2 cells overexpressing miR-146a-5p into the armpit of the right forelimb (n=5) of BALB/c nude mice. The lengths and widths of the solid tumors were measured every seven days. The tumor volume was calculated with the formula: tumor volume = length×width^2^/2.

### Luciferase assays

A luciferase reporter gene assay was performed in 293T cells to detect the binding between miR-146a-5p and the *HNRNPD* 3'UTR. A 1406-bp segment of the *HNRNPD* 3'UTR was cloned into the psi-CHECK2 vector (Promega, USA). TMECs (2×10^4^ cells/well) were incubated in 24-well plates for 24 h. Then, Lipofectamine 2000 (Invitrogen) was used to transfect the cells with either the control vector or the miR-146a-5p luciferase reporter gene vector. After 48 hours, the cells were lysed, the substrate was added, and luminescence was measured with a luciferase assay system on a GloMax bioluminescence detector (Promega, USA), in accordance with the manufacturer’s protocol.

### SiRNA for silencing HNRNPD

An RNA interference assay was performed to verify the function of HNRNPD. SiRNAs were purchased from Sigma-Aldrich, and the sequences are shown in Supplementary Table 2. TMEC1 and TMEC2 cells were seeded into six-well plates at a density of 5×10^4^ cells per well. The transfection experiment was carried out when the cells were 40% confluent. Lipofectamine™ and RNAiMAX were mixed into Opti-MEM, and the mixture was diluted to three graded concentrations: 25 nM, 50 nM and 100 nM. Six hours later, the transfection reagents were replaced with fresh medium. After 24 hours, qRT-PCR and Western blotting were used to determine the siRNA interference efficiency. An MTS assay was used to assess cell viability; flow cytometry was used to detect the cell cycle distribution and apoptosis; and a clonogenicity assay was performed as described previously [23].

### Overexpression of HNRNPD for the functional recovery assay

A pLenO-RIP plasmid vector, which carries a puromycin-resistant gene, was used to overexpress *HNRNPD* in TMEC1 cells according to the manufacturer’s instructions (Invabio Biotechnology, Shanghai, China). Then, CCK-8 and colony formation assays were performed as above.

### Statistical analysis

Each experiment was performed three times, and the data are presented as the mean ± standard deviation. Statistical significance was calculated with Student’s t-test for two-group comparisons. P < 0.05 was considered statistically significant. Data analyses were performed in GraphPad Prism 5.0 software.

### Ethics approval

The procedures of this study were approved by the Institutional Review Board of The Second Affiliated Hospital of Soochow University.

## Supplementary Material

Supplementary Figures

Supplementary Tables
