# Supplementary material for: Downregulation of miRNA-146a-5p promotes malignant transformation of mesenchymal stromal/stem cells by glioma stem-like cells
Source: Aging (Albany NY). 2020 May 25;12(10):9151–72. doi: 10.18632/aging.103185 (PMC7288935; doi:10.18632/aging.103185)
Supplement: Supplementary Figures [file aging-12-103185-s002..pdf]

SUPPLEMENTARY FIGURES

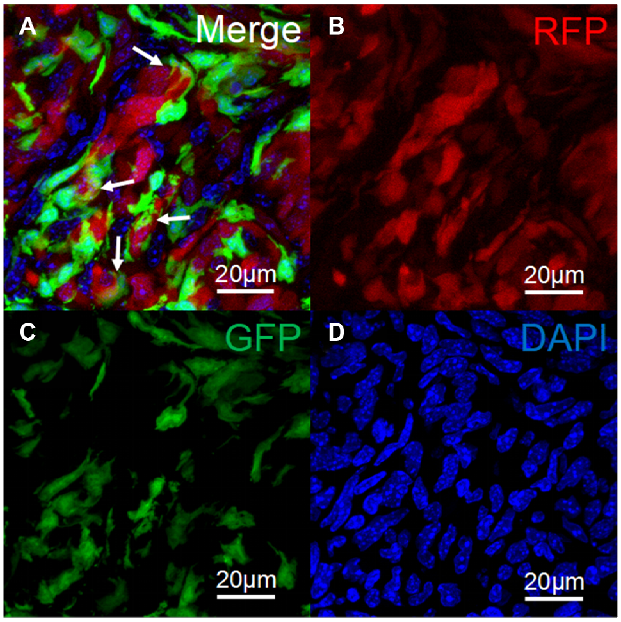

Supplementary Figure 1. Xenograft tumor of GSCs in chimeric mice (Model II). (**A**) Under confocal microscopy, close contact could be observed between the bone marrow-derived EGFP+ cells and RFP+ GSCs (white arrow); (**B**) RFP expressing GSCs under confocal microscopy; (**C**) GFP expressing bone marrow-derived EGFP+ cells; (**D**) DAPI labeled cell nucleus. Scale bar: 20 μm.

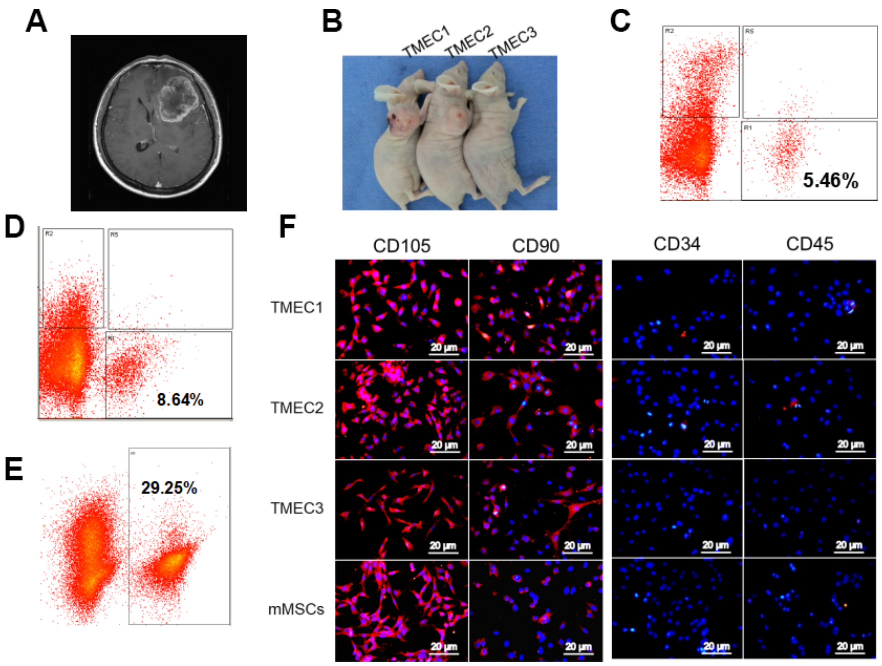

Supplementary Figure 2. (**A**) Magnetic resonance image of a 34-year-old male patient diagnosed with glioblastoma; (**B**) Tumorigenicity of the three transformed cell lines: TMEC1, TMEC2 and TMEC3; (**C**) Fluorescence-activated EGFP$^+$ cell sorting of the intracerebral xenograft of GSCs; (**D**) Fluorescence-activated EGFP$^+$ cell sorting of the intracerebral xenograft of clinical tumor specimens; (**E**) Immunofluorescence of the three tMSC lines. Scale bar: (**C**) 2 μm

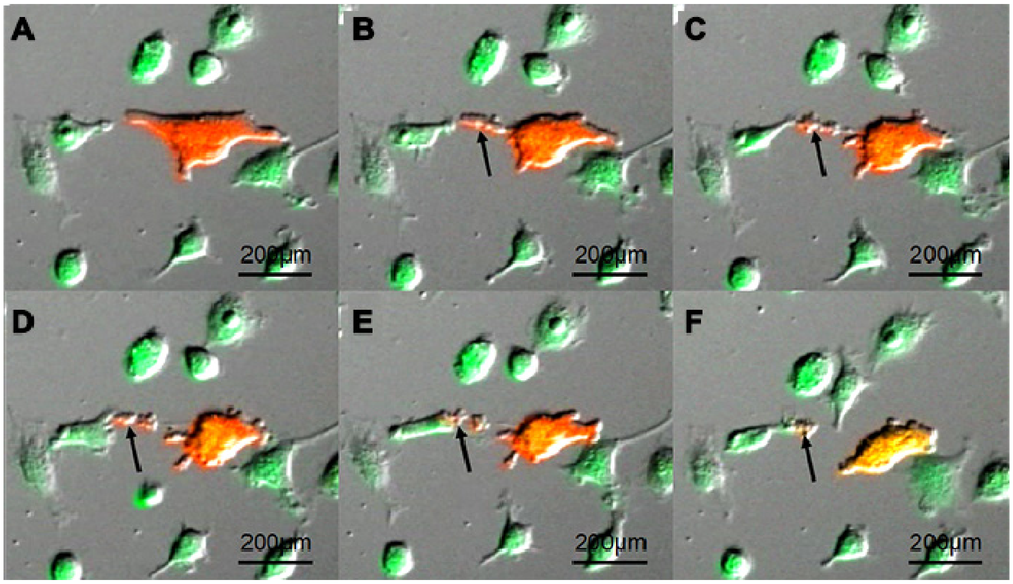

Supplementary Figure 3. Direct co-culture of GSCs and MSCs *in vitro*. (**A**) Time-lapse photography of a live-cell imaging station revealed tight contacts between RFP+ GSCs and EGFP+ MSCs; B-F) Both GSCs and MSCs protruded cell processes toward each other (**B**), which released vesicle-like mini-particles (**C**–**E**) and turned yellow (**F**) after the two kinds of particles had fused (black arrow). Scale bar: 200 µm.

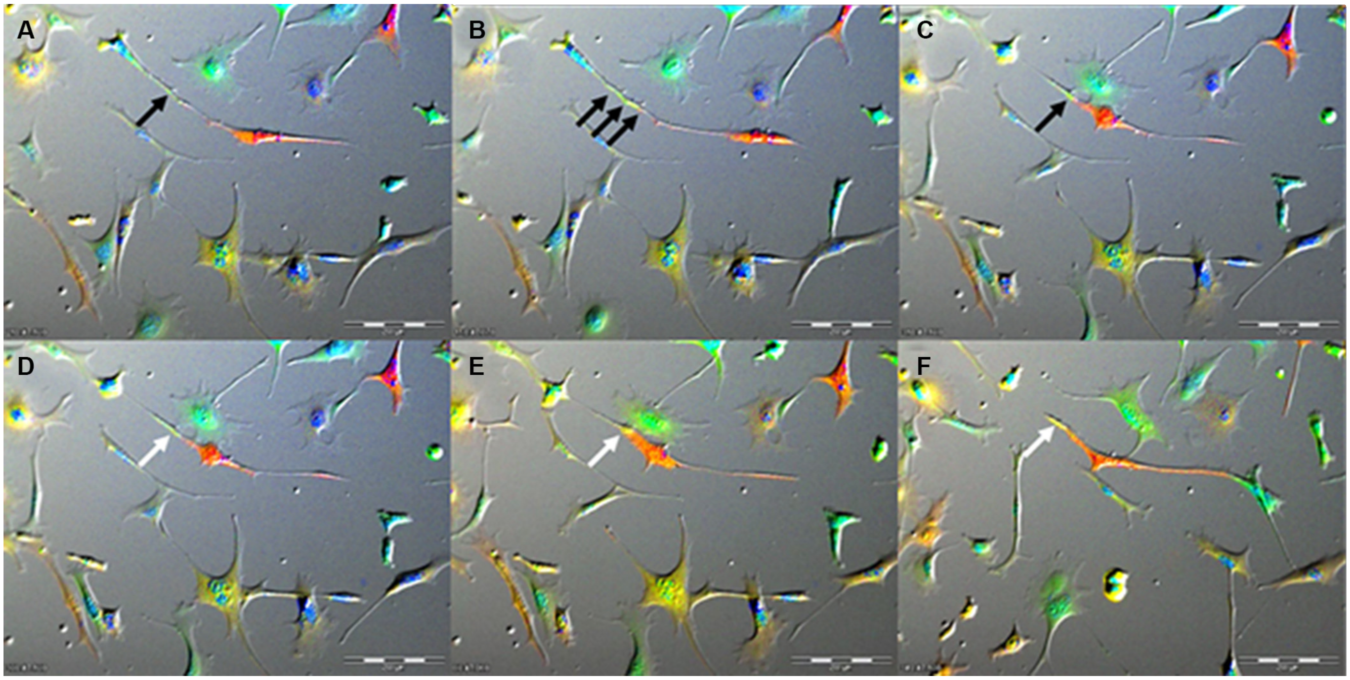

Supplementary Figure 4. GSCs and MSCs were co-cultured *in vitro*. RFP+ GSCs communicated with EGFP+ MSCs via long, tiny microtube-like structures (**A**–**C**, black arrow). After the intercellular cytoplasm exchange, these microtubes turned yellow (**D**–**F**, white arrow). Scale bar: 200 µm.

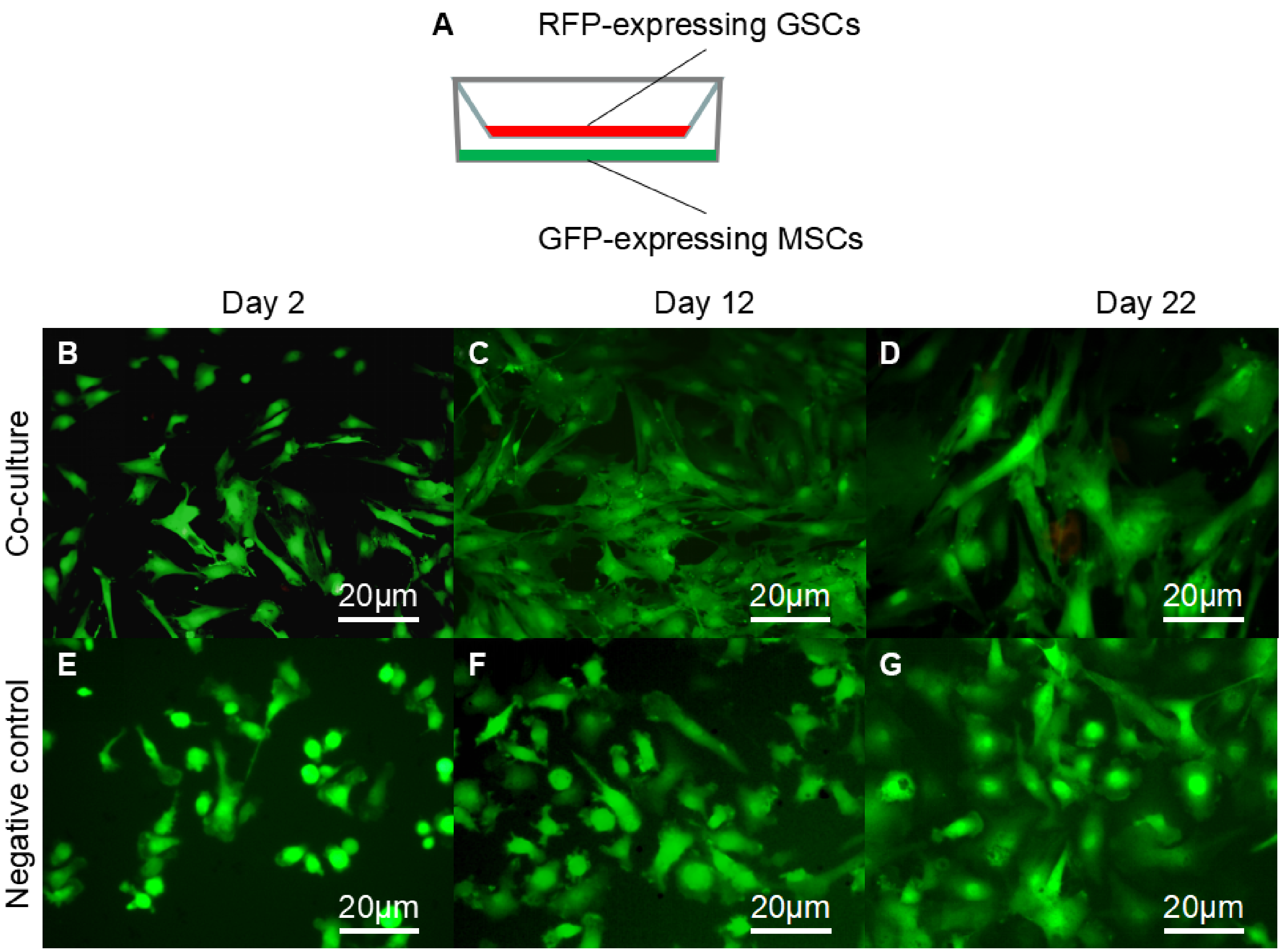

Supplementary Figure 5. Transwell indirect co-culture of GSCs and MSCs revealed certain morphological changes in MSCs, but no activation of MSC proliferation. (**A**) Schematic overview of indirect co-culture in Transwell chambers; (**B**) Day 2, no obvious proliferation of MSCs when indirectly co-cultured with GSCs, just certain morphological changes compared with MSCs cultured alone (**E**, control); (**C**) Day 12, MSC proliferation could be observed in both the co-culture and the control plate (**F**); (**D**) Day 22, the proliferation of MSCs decreased gradually, either when the cells were indirectly co-cultured with GSCs, or when they were cultured alone (**G**). Scale bar: 50 μm.

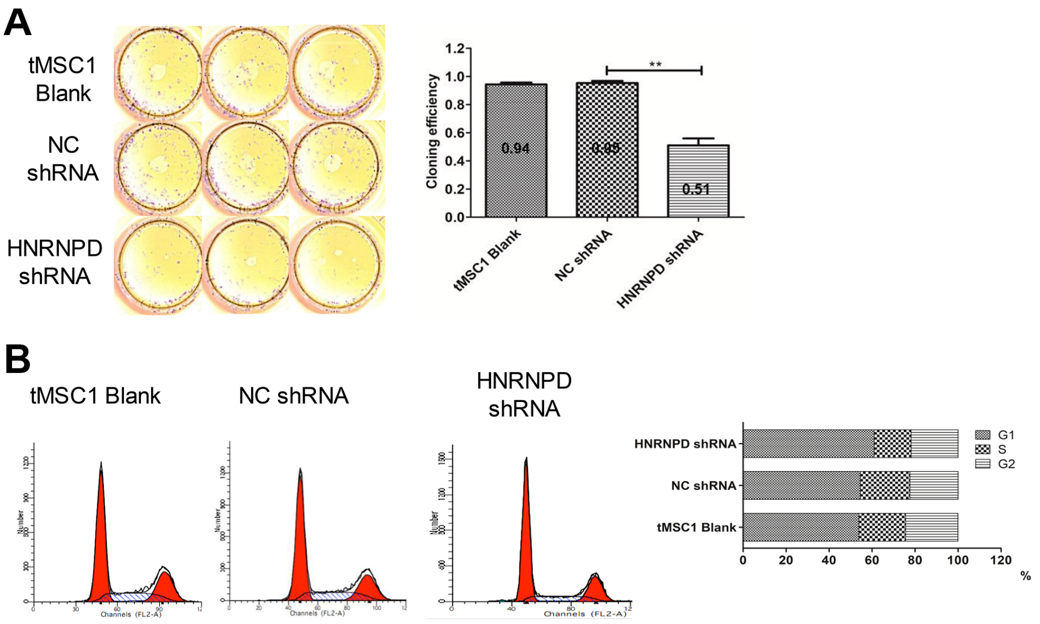

Supplementary Figure 6. (**A**) Clone formation assay with HNRNPD-overexpressing cells; (**B**) Flow cytometry assay detecting the cell cycle in HNRNPD-overexpressing cells.

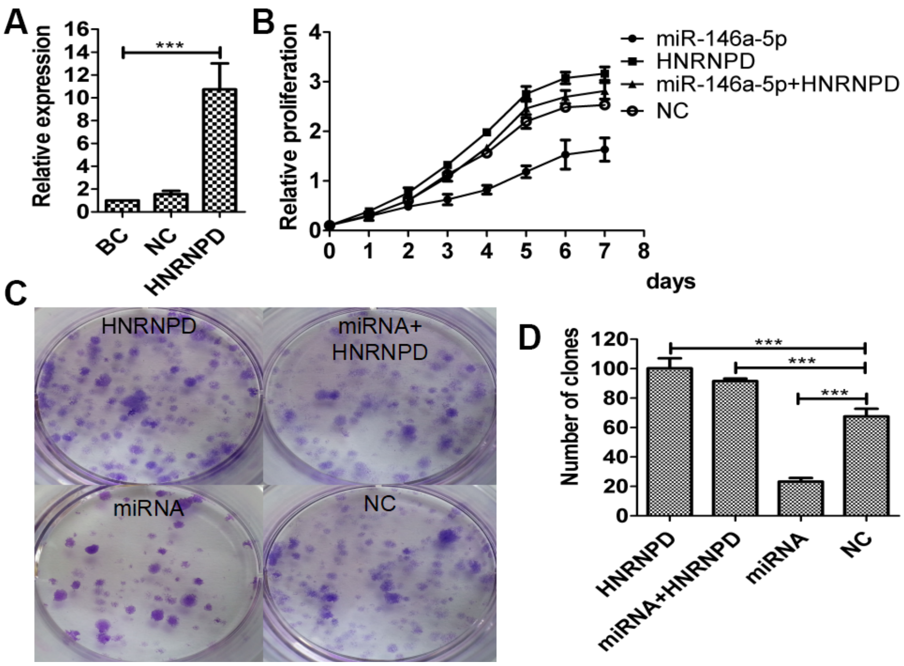

Supplementary Figure 7. Functional recovery assay. (**A**) qRT-PCR after overexpression of HNRNPD; (**B**) CCK-8 cell proliferation curve; (**C**) Colony formation assay of tMSC1 cells overexpressing HNRNPD and miRNA-146a-5p; (**D**) Statistical analysis of the colony formation assay.

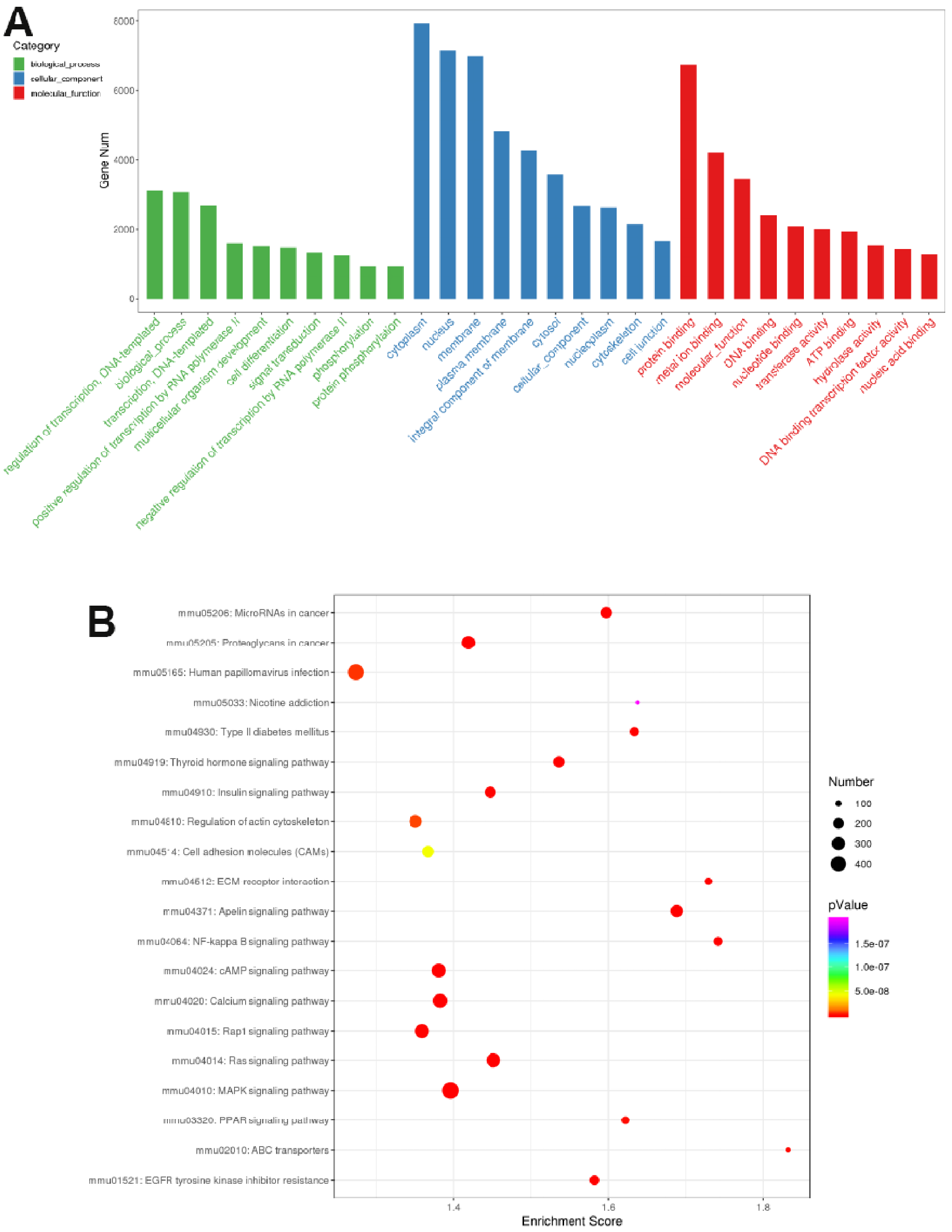

Supplementary Figure 8. GO and KEGG analyses of several dysregulated miRNAs. (**A**) Top 30 GO terms; (**B**) Top 20 KEGG pathway classifications.
