# Supplementary material for: Downregulation of miRNA-146a-5p promotes malignant transformation of mesenchymal stromal/stem cells by glioma stem-like cells
Source: Aging (Albany NY). 2020 May 25;12(10):9151–72. doi: 10.18632/aging.103185 (PMC7288935; doi:10.18632/aging.103185)
Supplement: Supplementary Tables [file aging-12-103185-s001..pdf]

SUPPLEMENTARY TABLES

Supplementary Table 1. Sequences of the miRNA and target gene primers.

		Sequences (5'-3')
Human β-actin	F	ACATCCGCAAAGACCTGTAC
	R	GCCATGCCAATCTCATCTTG
Mouse β-actin	F	CTTTGCAGCTCCTTCGTTG
	R	TGGTAACAATGCCATGTTCA
miR-27b-3p	miR-27-FO-2	TGGCAGTTCACAGTGGCTAAG
	miR-RE-6	CAGAGCAGGGTCCGAGGTA
miR-22-3p	miR-22-3p-FO-2	GCGGTCAAGCTGCCAGTT
	miR-RE-12a	TATGGTTGTTCACGACTCCTTCAC
miR-146a-5p	miR-146-FO	CTGCCGCTGAGAACTGAATT
	miR-RE-6	CAGAGCAGGGTCCGAGGTA
miR-146b-5p	miR-146b-FO-1	GATGCTGATGAGAACTGAATTCC
	mir-RE-7f	TATGGTTGTTCTGCTCACTGTCTC
miR-148-3p	miR-148a-3p-FO	CAGAGCTCTCAGTGCACTACAGA
	miR-RE-12a	TATGGTTGTTCACGACTCCTTCAC
miR-181a-5p	miR-181a-FO-1	TGCCGAACATTCAACGCT
	miR-RE-6	CAGAGCAGGGTCCGAGGTA
miR-181b-5p	miR-181b-FO-1	TCTGCCGAACATTCATTGCT
	miR-RE-6	CAGAGCAGGGTCCGAGGTA
miR-142-3p	miR-142-3p-FO-2	TGCTGCTGTGTAGTGTTTCCTACT
	miR-RE-12a	TATGGTTGTTCACGACTCCTTCAC
miR-486-5p	miR-486-5p–FO-2	GATGCACATCCTGTACTGAGCTG
	miR-RE-12a	TATGGTTGTTCACGACTCCTTCAC
HNRNPD	F	CATGCATTTACCCTGTTGAC
	R	TCCGTTTCCACCTTTATCAG
GAPDH	F	GGCCTCCAAGGAGTAAGAAA
	R	GCCCCTCCTGTTATTATGG

Supplementary Table 2. SiRNA sequence for *HNRNPD* and its negative control.

siRNA	Sequence:(5'-3')	Size	Epsilon 1/(mMcm)	MW g/mole	OD
SASI_Mm_00292473	CAAAUUUGGUGAAGUUGUAdTdT	21	217.9	6726	
SASI_Mm_00292473_AS	UACAACUUCACCAAAUUUGdTdT	21	205.3	6621	
	Duplex of above			13347	2.0
NC	UUCUCCGAACGUGUCACGUTT	21	196.2	6563	
NC_AS	ACGUGACACGUUCGGAGAATT	21	213.6	6712	
	Duplex of above			13275	1.0
